# Supplementary material for: HIVprotI: an integrated web based platform for prediction and design of HIV proteins inhibitors
Source: J Cheminform. 2018 Mar 9;10:12. doi: 10.1186/s13321-018-0266-y (PMC5845081; doi:10.1186/s13321-018-0266-y)
Supplement: Supplementary file 2 — Additional file 2. Source code of HIVProtI web server. [file 13321_2018_266_MOESM2_ESM.zip › HIVprotI_Source-code/hivprotI/weka-3-6-6/doc/weka/gui/explorer/Explorer.CapabilitiesFilterChangeEvent.html]

Explorer.CapabilitiesFilterChangeEvent


---


|  |  |  |
| --- | --- | --- |
| **Overview** \| **Package** \| **Class** \| **Tree** \| **Deprecated** \| **Index** \| **Help** \| **Weka's home** | |  |
| **PREV CLASS**   **NEXT CLASS** | **FRAMES**    **NO FRAMES**     **All Classes** |  |
| SUMMARY: NESTED \| FIELD \| CONSTR \| METHOD | DETAIL: FIELD \| CONSTR \| METHOD |  |


---


## weka.gui.explorer Class Explorer.CapabilitiesFilterChangeEvent

```
java.lang.Object
  java.util.EventObject
      javax.swing.event.ChangeEvent
          weka.gui.explorer.Explorer.CapabilitiesFilterChangeEvent
```

**All Implemented Interfaces:**: java.io.Serializable

**Enclosing class:**: Explorer

---

``` public static class Explorer.CapabilitiesFilterChangeEvent extends javax.swing.event.ChangeEvent ```

This event can be fired in case the capabilities filter got changed

**Version:**
:   $Revision: 7193 $

**Author:**
:   FracPete (fracpete at waikato dot ac dot nz)

**See Also:**: Serialized Form

---

**Constructor Summary**

`Explorer.CapabilitiesFilterChangeEvent(java.lang.Object source,
Capabilities filter)`
  
          Constructs a GOECapabilitiesFilterChangeEvent object.


| **Method Summary** | |
| --- | --- |
| `Capabilities` | `getFilter()`             returns the associated Capabilities filter |

**Methods inherited from class java.util.EventObject**

`getSource, toString`

**Methods inherited from class java.lang.Object**

`equals, getClass, hashCode, notify, notifyAll, wait, wait, wait`

**Constructor Detail**

### Explorer.CapabilitiesFilterChangeEvent

```
public Explorer.CapabilitiesFilterChangeEvent(java.lang.Object source,
                                              Capabilities filter)
```

:   Constructs a GOECapabilitiesFilterChangeEvent object.

    **Parameters:**: `source` - the Object that is the source of the event: `filter` - the responsible capabilities filter


**Method Detail**

### getFilter

```
public Capabilities getFilter()
```

:   returns the associated Capabilities filter

    :   **Returns:**: the filter


---


|  |  |  |
| --- | --- | --- |
| **Overview** \| **Package** \| **Class** \| **Tree** \| **Deprecated** \| **Index** \| **Help** \| **Weka's home** | |  |
| **PREV CLASS**   **NEXT CLASS** | **FRAMES**    **NO FRAMES**     **All Classes** |  |
| SUMMARY: NESTED \| FIELD \| CONSTR \| METHOD | DETAIL: FIELD \| CONSTR \| METHOD |  |


---
